# Microfluidic approaches for producing lipid-based nanoparticles for drug delivery applications

**DOI:** 10.1063/5.0150345

**Published:** 2023-09-18

**Authors:** Caterina Piunti, Elisa Cimetta

**Affiliations:** 1 Department of Industrial Engineering (DII), University of Padua—Via Marzolo 9, 35131 Padova, Italy; 2 Fondazione Istituto di Ricerca Pediatrica Città della Speranza (IRP)—Corso Stati Uniti 4, 35127 Padova, Italy

## Abstract

The importance of drug delivery for disease treatment is supported by a vast literature and increasing ongoing clinical studies. Several categories of nano-based drug delivery systems have been considered in recent years, among which lipid-based nanomedicines, both artificial and cell-derived, remain the most approved. The best artificial systems in terms of biocompatibility and low toxicity are liposomes, as they are composed of phospholipids and cholesterol, the main components of cell membranes. Extracellular vesicles—biological nanoparticles released from cells—while resembling liposomes in size, shape, and structure, have a more complex composition with up to hundreds of different types of lipids, proteins, and carbohydrates in their membranes, as well as an internal cargo. Although nanoparticle technologies have revolutionized drug delivery by enabling passive and active targeting, increased stability, improved solubilization capacity, and reduced dose and adverse effects, the clinical translation remains challenging due to manufacturing limitations such as laborious and time-consuming procedures and high batch-to-batch variability. A sea change occurred when microfluidic strategies were employed, offering advantages in terms of precise particle handling, simplified workflows, higher sensitivity and specificity, and good reproducibility and stability over bulk methods. This review examines scientific advances in the microfluidics-mediated production of lipid-based nanoparticles for therapeutic applications. We will discuss the preparation of liposomes using both hydrodynamic focusing of microfluidic flow and mixing by herringbone and staggered baffle micromixers. Then, an overview on microfluidic approaches for producing extracellular vesicles and extracellular vesicles-mimetics for therapeutic applications will describe microfluidic extrusion, surface engineering, sonication, electroporation, nanoporation, and mixing. Finally, we will outline the challenges, opportunities, and future directions of microfluidic investigation of lipid-based nanoparticles in the clinic.

## INTRODUCTION

Improving the effectiveness of drug delivery methods would significantly increase the efficacy of medical treatments. This necessitates the development of novel strategies that can optimize the bioavailability and specificity of therapeutic agents while simultaneously minimizing their potential toxicity. Conventional approaches to drug administration often face challenges such as limited drug solubility, poor tissue penetration, and nonspecific distribution, which can significantly impact the therapeutic outcome (Shi *et al.*, 2016). Efficient drug delivery systems (DDSs) aim to overcome these obstacles by precisely targeting the desired site of action, maximizing drug absorption and retention, and minimizing adverse effects on healthy tissues. Following groundbreaking discoveries in the handling and use of nanoscaled materials, several nano-based DDSs are being considered for drug-targeting applications (Vargason *et al.*, 2021). Among all nanoparticles (NPs) for DDSs, lipid-based NPs including liposomes and extracellular vesicles (EVs) have shown great potential. Both liposomes and EVs are composed of one lipid bilayer, can have a size between 50 and 120 nm, and can be loaded with lipophilic and hydrophilic drugs. Their main advantages and disadvantages are summarized in Table I.

**TABLE I.** Comparison between liposomes and EVs as drug vehicles.

	Advantages	Disadvantages
Liposomes	Prolonged circulation if PEGylated (Klibanovl *et al.*, 1990) Possible affinity with targeting ligands (van der Koog *et al.*, 2022) Well-established handling techniques (Armstrong *et al.*, 2017) Precise control over contents (van der Koog *et al.*, 2022)	Rapid clearance from the bloodstream (Gregoriadis and Ryman, 1972) Low targeting efficiency (Sercombe *et al.*, 2015) High immunogenicity (Murphy *et al.*, 2019)
EVs	Prolonged circulation if PEGylated (Kooijmans *et al.*, 2016) High organotropism (Hoshino *et al.*, 2015) Innate biocompatibility and complex biological composition (Armstrong *et al.*, 2017) High targeting efficiency (Lösche *et al.*, 2009) Intrinsic ability to cross tissue and cellular barriers (Alvarez-Erviti *et al.*, 2011)	Moderate clearance from the bloodstream (Matsumoto *et al.*, 2020) Low production yield (Goh *et al.*, 2017) Low loading efficiency (Donoso-Quezada *et al.*, 2020)

The landscape of FDA-approved NPs is dominated by liposomes, which consist of a spherical bilayer formed by up to four lipid types with an internal aqueous core, encapsulating up to two therapeutic agents (Anselmo and Mitragotri, 2019). Having gained clinical approval as the first nanocarriers in 1995, liposomes emerged as the most studied and successful DDSs for a wide range of applications including chemotherapy, gene therapy, and vaccination (Bobo *et al.*, 2016). The advantages of liposomes are several, including high stability, efficient drug loading capacity, and a large surface area owing to their small particle size. Compared to other drug carriers like polymeric NPs, liposomes exhibit superior biocompatibility. Moreover, liposomes possess the ability to accommodate hydrophobic and hydrophilic compounds within their lipid bilayer and aqueous core, respectively, while their surface can be tailored with specific ligands to enable immune evasion and targeted delivery (van der Koog *et al.*, 2022). Nonetheless, unmodified liposomes are readily recognized by the mononuclear phagocytic system and quickly cleared from the bloodstream. To address this limitation, many clinically approved synthetic NPs are functionalized with polyethylene glycol (PEG), which reduces macrophage uptake and significantly prolongs the circulation half-life from hours to several days (Guimarães *et al.*, 2021).

A notable breakthrough in the field of DDSs also occurred with the discovery of EVs and the recognition of their role as mediators of intercellular communication (Théry *et al.*, 2002). EVs bear similarities to liposomes in terms of their size, shape, and overall structure, but possess more complex bilayers composed of hundreds of different lipid, protein, and carbohydrate species, as well as cargo and surface-associated molecules (Witwer and Wolfram, 2021). Secreted by all living cells, EVs can be purified from the conditioned medium of cultured cells, biological tissues, or various bodily fluids (van Niel *et al.*, 2018). Upon release, EVs can either bind to nearby cells or the extracellular matrix or disseminate through body fluids. This remarkable mobility enables EVs to deliver vital contents and signals to cells both locally and at distant sites. Notably, upon intravenous injection, labeled EVs swiftly reach tissues throughout the body within minutes, positioning them as one of the fastest delivery vehicles known (Robbins and Morelli, 2014). Given their cellular origin, EVs hold immense promise as naturally targeted and personalized DDSs, amenable of being engineered with therapeutic agents (Walker *et al.*, 2019).

Given their relatively recent history and intrinsic heterogeneity, there are still numerous aspects of the fundamental biology of EVs that require further investigations. EV preparations consist of diverse subtypes, exhibiting variations in terms of their subcellular origins, sizes, and surface protein markers. It is plausible that exploring specific subtypes for therapeutic purpose may lead to the identification of additional beneficial modifications or strategies for the advancement of EV-based therapeutics.

EVs demonstrate excellent biocompatibility and stability, with minimal immunogenicity, and have been found to support tissue regeneration and stimulate specific immune responses (El Andaloussi *et al.*, 2013). Additionally, EVs exhibit improved targeting of tumors and reduced cytotoxic effects (Murphy *et al.*, 2019).

Despite the distinct and more complex composition of EVs in comparison to liposomes, their circulation time is not consistently superior. While Kamerkar *et al.* reported that EVs derived from a commercial source were detectable in the circulation 24 h after intraperitoneal injection (Kamerkar *et al.*, 2017), recent evidence suggests that the prolonged presence of EVs in mouse models' circulation is unlikely. For instance, Lázaro-Ibáñez *et al.* reported a half-life of EVs in mouse circulation to be less than 10 min (Lázaro-Ibánez *et al.*, 2021). However, it has been demonstrated that PEGylation can also enhance the circulation time of EVs while preserving their ability to effectively deliver cargo to specific cell types (Kooijmans *et al.*, 2016).

Although PEGylation is the gold standard to enhance lipid-based NPs stability, it is accompanied by the potential risk of hypersensitivity reactions and accelerated blood clearance due to the presence of pre-existing or newly formed anti-PEG antibodies. A recent study by Estapé Senti *et al.* revealed that complement activation induced by anti-PEG antibodies can compromise the integrity of the NP's bilayer or surface, resulting in poorly controlled and earlier drug release. These observations emphasize the importance of the potential impact of anti-PEG antibodies on nanoparticle behavior and drug delivery efficacy (Estapé Senti *et al.*, 2022).

Like liposomes, EVs can encapsulate both hydrophobic and hydrophilic contents, whether in their lipid bilayer or internal compartment. EVs can also easily cross tissue “roadblocks” including the blood–brain and blood–tumor barriers (Alvarez-Erviti *et al.*, 2011), which have historically posed challenges in drug delivery (Nagelkerke *et al.*, 2021). EVs' practical application as DDSs has however been mainly hindered by the challenges associated with their limited production yield. The generation of a sufficient quantity of EVs requires a substantial amount of starting materials, including cells and culture medium, and involves laborious isolation techniques with relatively low yields.

In recent years, the development of EVs-mimetics has gained attention as a potential solution to address the limitations of conventional EVs isolation and purification methods. EVs-mimetics can be classified into two main categories: synthetic EVs-mimetics and cell-derived nanovesicles (CDNs) (Antimisiaris *et al.*, 2018). These artificial EVs-mimetics are created by utilizing synthetic or semi-synthetic materials as starting components, and since natural EVs adopt spherical lipid bilayer structures, liposomes serve as a logical foundation for their creation. Through detailed analyses of the lipid, protein, and nucleic acid composition of EVs and considering that not all components found in natural EVs are essential for their activity, only functional elements can be selectively incorporated into EVs mimetics (Kooijmans *et al.*, 2012). On the other hand, CDNs are generated using cellular components other than EVs, such as whole cells. The process of producing CDNs involves a physical method that results in the formation of nano-sized vesicles while preserving the original protein configuration of the parent cells. Similar to EVs, CDNs maintain characteristics that promote efficient cellular uptake: the preserved surface proteins enable innate targeting abilities, allowing for selective delivery to specific cell types (Goh *et al.*, 2017).

Overall, this unique set of properties makes EVs and EVs-mimetics highly promising for enhancing the effectiveness of cancer therapies (Witwer and Wolfram, 2021).

However, the precise manipulation and manufacturing of lipid-based NPs for DDS applications encounter several hurdles including labor-intensive and time-consuming procedures. Conventional methods of synthesizing liposomes and engineering EVs such as bulk extrusion and bulk mixing, have poor control over particle size and polydispersity, inconsistent batch to batch reproducibility, and pose challenges in scaling up production (Tiboni *et al.*, 2021).

Microfluidics, the science of precise manipulation of fluids at the micrometer scale, has emerged as a promising technique to advance NPs research by improving product quality and reproducibility (Sackmann *et al.*, 2014). Microfluidic systems offer several advantages over traditional methods as they are highly customizable, automatable, and scalable. They enable highly efficient mass transport, leading to rapid synthesis, high control over NP structure, and enhanced reproducibility (Damiati *et al.*, 2018). The precise liquid handling and mixing capabilities of microfluidics make it an ideal platform for engineering nano-vesicles and EVs (Maeki *et al.*, 2018). Flow in microscaled channels and chambers follows low-Reynolds laminar profiles, and molecular diffusion dominates over mass transport phenomena allowing precise tuning of concentration patterns between streams containing different species. Mixing within microfluidic channels enhances reaction rates and can be integrated with sample processing methods and molecular analyses (Squires and Quake, 2005). Finally, microfluidic devices allow for continuous flow operations, ensuring consistent NPs production quality over time, crucial in the pharmaceutical industry (Osouli-Bostanabad *et al.*, 2022).

This review aims to explore representative advances, challenges, opportunities, and future directions in the manufacturing of lipid-based nanomedicines using microfluidics, with a specific focus on liposomes, EVs, and EVs-mimetics and their applications in drug delivery. Table II summarizes key characteristics of the examined devices and techniques: hydrodynamic flow focusing (HFF), T-mixers, staggered herringbone micromixers (SHM), invasive lipid nanoparticle production (iLiNP), extrusion, surface engineering, sonication, electroporation, and nanoporation.

**TABLE II.** Summary of recent studies on the production of liposomes and EVs for drug delivery applications using microfluidic platforms. AO: acridine orange hydrochloride; AT: atenolol; BSA: bovine serum albumin; CBD: cannabidiol; Chol: cholesterol; CoQ_10_: coenzyme Q10; DC: 3β-[N-(dimethylaminoethane)-carbamoyl]; DCP: dihexadecylphosphate; DiO: cell-labeling solution; DMG-PEG2000: 1,2-dimyristoyl-sn-glycerol, methoxy polyethylene glycol 2000; DMPC: 1,2-dimyristoyl-sn-glycero-3-phosphocholine; DOPC: 1,2-dioleoyl- sn-glycero-3-phosphocholine; DOPE: 1,2-dioleoyl-sn-glycero-3-phosphoethanolamine; DOPS: 1,2-dioleoyl-sn-glycero-3-phospho-L-serine; DOX: doxorubicin; DPPC: 1,2-dihexadecanoyl-sn-glycero-3-phosphocholine; DSPC: 1,2-disteroyl-sn-glycero-3-phosphocholine; DSPE-PEG2000-Folate: 1,2-distearoyl-sn-glycero-3-phosphoethanolamine-N-[folate(polyethylene glycol)-2000]; ES: embryonic stem; EV: extracellular vesicles; FA: folic acid; HFF: hydrodynamic flow focusing; iLiNP: invasive lipid nanoparticle production; MEFs: mouse embryonic fibroblasts; NPs: nanoparticles; NV: nanovesicles; PC: phosphatidylcholine; PEG: polyethylene glycol; PEG2000-PE: 1,2-distearoyl-sn-glycero-3-phosphoethanolamine-N-[folate(polyethylene glycol)-2000; PEG5000-PE: 1,2-dimyristoyl-sn-glycero-3-phosphoethanolamine-N-[methoxy(polyethylene glycol)-5000]; PLGA: poly(lactic-co-glycolic acid); POPC: 1-palmitoyl-2-oleoyl-sn-glycero-3-phosphocholine; Q: quinine; RhB: rhodamine B; SM: sphingomyelin; SPC: phospholipon 90-G (soybean lecithin, phosphatidylcholine); TAT: cell penetrating peptide; YSK05: pH-sensitive cationic lipid; ZIF-8: zeolitic imidazolate framework-8.

	Microfluidic technique	Size (nm)	Loading capacity	Lipid composition/EV and NV parent cells	Modification\cargos	Throughput	References
Liposomes	HFF	80–110	N.A.	DMPC/Chol/DCP/PEG5000-PE/PEG2000-PE/DSPE-PEG2000-Folate	PEG- and folate- conjugated	N.A.	Hood *et al.* (2013)
	HFF	190	70%	DMPC/Chol/PEG2000-PE	DOX and AO	10^10^–10^12^ liposomes ml^−1^	Hood *et al.* (2014)
	HFF	107	75%	DC-Chol/PC/DSPE-PEG	Antisense Bcl-2	N.A.	Koh *et al.* (2010)
	HFF	70	62%–74%	DMPC/Chol/DSPE-PEG2000	FA and TAT	N.A.	Ran *et al.* (2018)
	HFF	80–120	N.A.	DMPC/Cholesterol/PEG2000-PE	N.A.	∼ 100 mg h^−1^	Hood and Devoe, (2015)
	HFF	150	N.A.	N.A.	N.A.	1 kg day^−1^	Romanowsky *et al.* (2012)
	T-mixers	<150	∼80%	SPC/Chol	CBD	N.A.	Tiboni *et al.* (2021)
	SHM	20–140	∼100%	POPC/Chol	DOX	N.A.	Zhigaltsev *et al.* (2012)
	SHM	40–50	50 mol%	PC/Chol	Propofol	N.A.	Kastner *et al.* (2015)
	SHM	50–100	20%–40%	PC, DMPC, DPPC, DSPC/Chol	Metformin and glipizide	N.A.	Joshi *et al.* (2016)
	SHM	70–200	70%–100%	DMPC/DSPC/Chol	AT and Q	N.A.	Guimarães Sá Correia *et al.* (2017)
	SHM	94–118	90%	DPPC/DSPC/DOPC/Chol	Leukocytes-derived membrane proteins	N.A.	Molinaro *et al.* (2018)
	SHM	120	17wt. %	DMPC/DPPC/DSPC	Curcumin	N.A.	Hamano *et al.* (2019)
	SHM	60–100	20%–35%	DMPC:Chol	Proteins	N.A.	Forbes *et al.* (2019)
	iLiNP	20–100	>90%	YSK05/Chol/DMG-PEG2000	FVII siRNA	N.A.	Kimura *et al.* (2018)
	iLiNP	50	∼70%	DOPE/SM/DMG-PEG2000	CoQ_10_	400 *μ*l 0.8 min^−1^	Hibino *et al.* (2019)
EVs/EVs-mimetics	Extrusion	60–120	20%	Murine ES cells	EVs-derived mRNAs and proteins	N.A.	Jo *et al.* (2014)
	Extrusion	100–300	30%	Murine ES cells	Fluorescent beads	∼150 × 10^8^ EVs per 10^6^ cells	Yoon *et al.* (2015)
	Surface Engineering	50–200	N.A.	Leukocytes	Tumor antigenic peptides	∼2 h for processing 4 10^4^ seeded cells	Zhao *et al.* (2019)
	Sonication	177.4	91%	Human lung cancer cells A549	EVs membrane-coated PLGA NPs loaded with DiO	N.A.	Liu *et al.* (2019)
	Sonication	116.2	N.A.	Human lung cancer cells A549	EVs membrane-coated ZIF-8 NPs loaded with FITC-BSA and RhB	N.A.	Lv *et al.* (2021)
	Electroporation	70–110	2–10 mRNA copies per EV	MEFs	Therapeutic mRNAs	10^12^ EVs per 10^6^ cells 2–3 day^−1^	Yang *et al.* (2020)
	Nanoporation	170 nm	4.9 *μ*g per 10^8^ EVs	Human lung cancer cells A549	DOX	8.5 10^8^ EVs ml^−1^ at 8 *μ*l min^−1^	Hao *et al.* (2021)
	iLiNP	90–121	>50%	DOPC/SM/Chol/DOPS/DOPE	siRNA	N.A.	Kimura *et al.* (2021)

## MICROFLUIDIC-BASED LIPOSOME PRODUCTION

The formation of lipid-based NPs such as liposomes relies on the diffusion of molecular species like alcohols, water, and lipids at the interface between the solvent and nonsolvent phases. Traditional methods of liposomes preparation, such as hydration of a phospholipid film and solvent vaporization, have limitations when it comes to industrial mass production due to the use of large quantities of volatile organic solvents, complex scaling-up processes, poor reproducibility, high cost, and multiple time-consuming steps (Liu *et al.*, 2022). The hydration technique, commonly used for manufacturing large multi- and unilamellar vesicles (100–1000 nm), involves the formation of a lipid film through the evaporation of an organic solvent from a lipid–solvent solution (Meure *et al.*, 2008). An aqueous phase is added to form multilamellar vesicles, which can be transformed into small unilamellar vesicles (<100 nm) through additional processes like sonication and extrusion (Koh *et al.*, 2010). To avoid the high labor-intensive steps of the hydration technique, the alternative solvent vaporization is frequently used for industrial-scale production of liposomes. It involves injecting a lipid suspension (for either hydrophobic or hydrophilic organic solvents) into a water phase. For example, with the ethanol injection method, lipids dissolved in ethanol are injected into the water phase while stirring, and then the solvent is removed (Pons *et al.*, 1993), resulting in vesicles ranging from 30 to 170 nm in size. Drawbacks, however, include poor encapsulation efficiency of hydrophilic compounds, low lipid solubility in ethanol, and low concentrations of lipids in the final solution due to the high ethanol content (Charcosset *et al.*, 2015). Liposomes obtained through these conventional methods typically undergo freeze/thaw cycles for drug loading, and possible additional post-processing functionalization, such as conjugations to add targeting ligands or imaging molecules.

The advantages of adopting microfluidic approaches include one-step fabrication of nearly monodispersed liposomes with adjustable diameters and surface properties by tuning flow rates, device geometries, solvent polarities, and concentrations and compositions of the lipid precursors (Shah *et al.*, 2019). Moreover, microfluidic-produced drug-loaded liposomes have higher encapsulation efficiency and more uniform and smaller size compared to those synthesized using traditional methods (Elsana *et al.*, 2019).

Various microfluidic approaches have been developed for the rapid and reproducible formation of drug-loaded liposomes, including hydrodynamic flow focusing (HFF) (Jahn *et al.*, 2004), micromixer-based (van Swaay and deMello, 2013), droplet-based (Deshpande *et al.*, 2016) (Davies *et al.*, 2012), tangential flow filtration (Dimov *et al.*, 2017), millimeter-scale flow reactors (Yanar *et al.*, 2020), and microfluidic jetting technique (Stachowiak *et al.*, 2008).

**FIG. 1.**
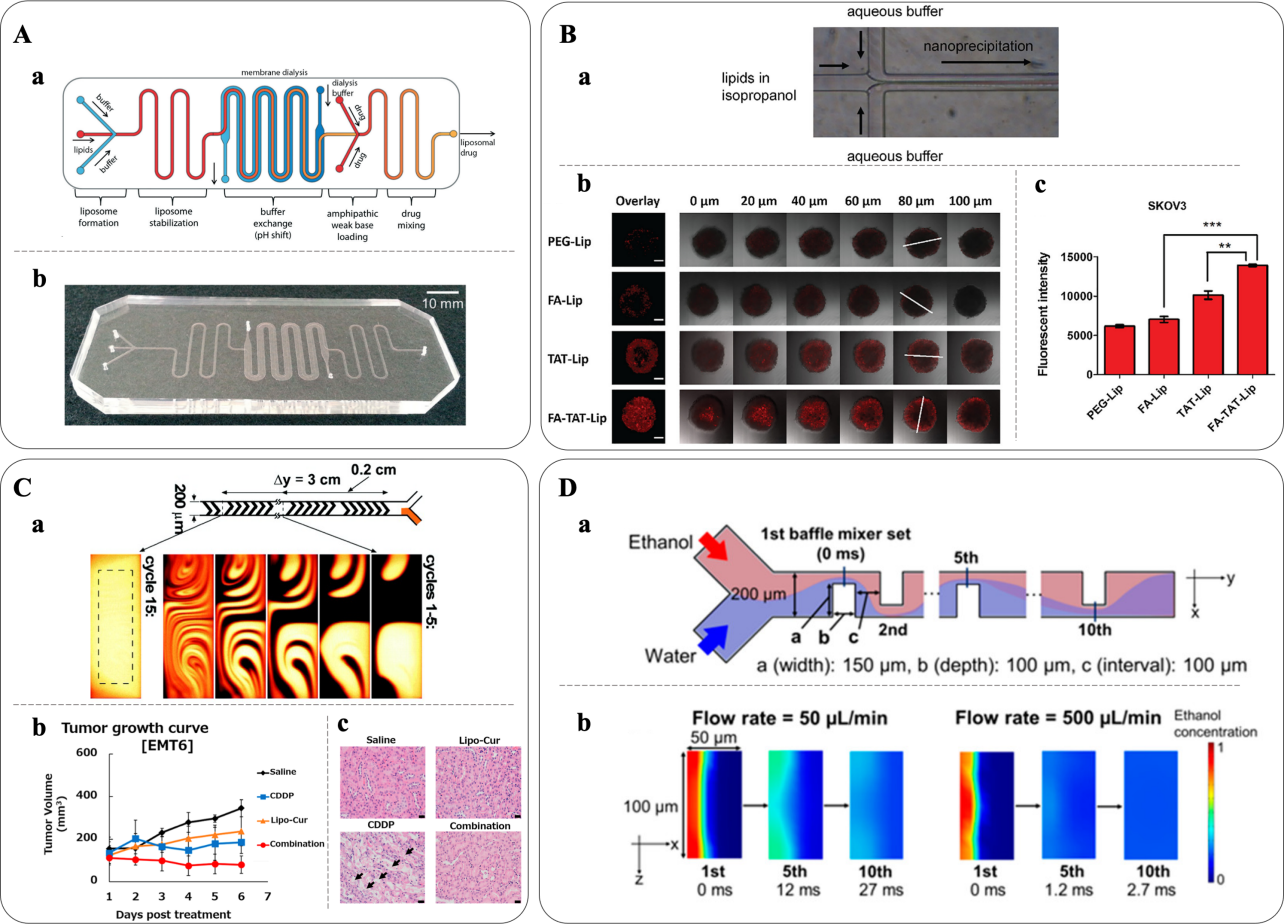
Microfluidic-based liposome production. (A-a) Schematic of a fully integrated microfluidic device made of PDMS and cellulose for liposome synthesis using HFF, buffer exchange via microdialysis and drug loading and (A-b) photograph of the fabricated device. Reproduced with permission from Hood *et al.*, Lab Chip **14**(17), 3359–3367 (2014). Copyright 2014 Royal Society of Chemistry. (B) Liposome formation through nanoprecipitation. (B-a) Schematic of a device. Liposomes form through self-assembly when the lipid solution is met with the aqueous buffer from the adjacent channels. (B-b) Fluorescently labeled liposomes with functional groups (PEG-Lip, FA-Lip, TAT-Lip, and FA-TAT-Lip) produced using an HFF-based device and tested on SKOV3 tumor spheroids; scale bars: 200 *μ*m. (B-c) Flow cytometry results demonstrated an increased uptake by the FA-TAT-Lip of 37% and 98% compared to the single ligand TAT-Lip and FA-Lip, respectively. Reproduced with permission from Ran *et al.*, Eur. J. Pharm. Biopharm. **130**, 1–10 (2018). Copyright 2018 Elsevier. (C-a) Liposomal curcumins (Lipo-Cur) were prepared by a microfluidic platform equipped with a staggered herringbone micromixer. Reproduced with permission from Stroock *et al.*, Science **295**(5555), 647–651 (2002). Copyright 2002 The American Association for the Advancement of Science. (C-b) Lipo-Cur antitumor activity was evaluated in tumor models in mice with EMT6 murine breast tumor cells inoculated into BALB/c mice. 7 days post tumor inoculation, the mice received an injection of either saline, free cisplatin (CDDP), Lipo-Cur, or combination of CDDP and Lipo-Cur. The combination treatment displayed enhanced effect as demonstrated by tumor growth kinetics. Lipo-Cur had also a protective effect against CDDP-induced kidney toxicity. (C-c) The kidney isolated from BALB/C mice was treated with either saline, Lipo-Cur, CDDP, or combination of Lipo-Cur and CDDP. CDDP induced significant acute tubular necrosis as indicated by the arrows, while the kidney histology was normal in other groups. Reproduced with permission from Hamano *et al.*, Mol. Pharm. **16**(9), 3957–3967 (2019). Copyright 2019 American Chemical Society. (D-a) Schematic of the iLiNP device featuring a two-dimensional baffle mixer enabling liposomes production and precise size tuning within a range of 20–100 nm, with intervals as small as 10 nm. (D-b) Computational fluid dynamic simulation of ethanol dilution in the iLiNP device at different flow rates demonstrated that the dilution performance was dramatically accelerated at 500 *μ*l/min, and ethanol was completely diluted within 3 ms. Reproduced with permission from Kimura *et al.*, ACS Omega, **3**(5), 5044–5051 (2018). Copyright 2018 American Chemical Society.

In this section, the focus is on the use of HFF and micromixer-based methods for the preparation of 20- to 100-nm size-controlled liposomes for applications as carriers in DDSs. Relevant examples are reported in Fig. 1.

## HYDRODYNAMIC FLOW FOCUSING

HFF is a microfluidic technique that represents a rapid, simple, and cost-effective approach for producing multifunctional liposomes with tailored surface modifications. In HFF, a solution containing lipids in alcohol is hydrodynamically directed into a narrow stream by aqueous buffer solutions. As the alcohol diffuses into the buffer, the lipids gradually assemble into planar bilayers, which then undergo bending to reduce the exposure of hydrophobic chains to the polar buffer. Eventually, planar bilayers close to form spherical liposomes with the bilayer separating an aqueous interior from an aqueous exterior. By adjusting factors such as lipid concentration, flow rate ratio (FRR) between the lipid- and water-phase streams, and total flow rate (TFR), the properties of the liposomes, including size, charge, and surface chemistry can be controlled (Zook and Vreeland, 2010).

The use of HFF for liposome synthesis was first demonstrated by Jahn *et al.* in 2004, who achieved size control ranging from 100 to 300 nm by adjusting flow velocity and FRR (Jahn *et al.*, 2004). Subsequent studies highlighted the advantages of microfluidic approaches in obtaining liposomes with surface modifications. Hood *et al.* synthesized nearly monodisperse PEG-modified and folate receptor-targeted liposomes (Hood *et al.*, 2013). Devoe's group efficiently loaded liposomes with amphipathic drugs such as doxorubicin (DOX) and acridine orange hydrochloride (AO), reducing the preparation and loading time from days to minutes [Fig. 1(a)]. Their platform integrated liposome formation, transmembrane ion gradient establishment using membrane dialysis, drug loading in a micromixer with structures enhancing interactions between liposomes and amphipathic compounds, and incubation. The encapsulation efficiency was ∼72% and ∼70% for DOX and AO, respectively (Hood *et al.*, 2014). Koh *et al.* improved upon Bcl-2 antisense deoxyoligonucleotides encapsulation compared to conventional methods using a five-inlet microfluidic HFF system (Koh *et al.*, 2010). Ran *et al.* used HFF to prepare a library of dual-ligand PEGylated liposomes with folic acid (FA) for tumor targeting, and TAT as cell-penetrating peptide for cell membrane translocation of the liposomes [Fig. 1(b)], demonstrating improved targeting and extended retention in various biological models, including a two-dimensional (2D) cell monolayer, three-dimensional (3D) tumor spheroid models, and a tumor-bearing mouse model. Their work allowed the combinatorial synthesis of libraries of liposomes with systematically varied properties, including size, zeta potential, targeting ligand, ligand density, and ligand ratio, and offered a new strategy for the identification of the best formulation with the optimal biological functions (Ran *et al.*, 2018).

The HFF method is amenable to scale up and adaptation to massive clinical applications and industrial manufacture, surpassing the limitation of other microfluidic-based methods.

Hood *et al.* reported high throughput and large-scale liposome production using a high aspect ratio vertical HFF device, increasing the production rate as high as 96 mg h^−1^, approximately 1000 times higher than using the original HFF device. However, the size of the formed LNPs was limited and ranged from 80 to 200 nm (Hood and Devoe, 2015). Parallel integrated microfluidic devices have also been explored to further enhance production throughput (Romanowsky *et al.*, 2012).

While HFF devices may not have been as widely employed as other microfluidic platforms, they offer significant advantages over traditional manufacturing methods in terms of cost-effectiveness and improved control over liposome properties.

## MICROMIXER-BASED METHOD

Micromixer-based devices improve on the throughput of liposome production by enhancing the mixing efficiency in the low-Reynolds, laminar flow conditions, where mass transfer occurs through passive molecular advection and diffusion (Whitehead *et al.*, 2009).

T-mixers are characterized by simple geometries and have been traditionally used for rapid mixing of lipid-based nanomedicines where an anti-solvent and solvent are combined under laminar flow, allowing diffusion-based mixing to occur at their interface (Camarri *et al.*, 2020). Recent studies demonstrated the ability to produce cannabidiol (CBD)-loaded liposomes through passive mixing using 3D fused deposition modeling-printed polypropylene T-mixers with zigzag bas-relief or a split-and-recombine channel shape. These T-mixers have shown the capability to generate liposomes smaller than 150 nm with high loading efficiency (Tiboni *et al.*, 2021).

However, T-mixers alone often result in limited control over particle size and typically require a large volume of starting solutions, which restricts their applicability in experimental studies. In this section, we focus on microfluidic devices that incorporate improved staggered herringbone micromixers (SHM) and baffle micromixers to produce liposomes for DDS applications.

## STAGGERED HERRINGBONE MICROMIXERS

SHM induce chaotic advection within microchannels, increasing mass transfer under laminar flow conditions and ultimately facilitating liposome production. The dimensions of the micromixer structures, including width, depth, and height, are crucial parameters of the mixing efficiency (Stroock *et al.*, 2002). SHM devices can be combined with the NanoAssemblr^TM^ (NA, Precision NanoSystems Inc., Vancouver, Canada) automated mixing platform for the reproducible, tunable, cost-effective, and scalable manufacture of lipid NPs. The NA platform consists of a benchtop instrument controlling the fluid flow through the SHM device (Belliveau *et al.*, 2012). Zhigaltsev *et al.* produced liposomes by mixing lipids (1-Palmitoyl-2-oleoyl-sn-glycero-3-phosphocholine and cholesterol) dissolved in ethanol with an aqueous stream using a SHM microfluidic device. The liposome size could be tuned from 20 to 140 nm by adjusting the flow rate and the FRR and could be efficiently loaded with DOX, showcasing their potential as drug delivery agents (Zhigaltsev *et al.*, 2012). Kastner *et al.* similarly generated size-controlled liposomes loaded with poorly soluble drugs in a high-throughput setting achieving high drug loading (50 mol%) of propofol, resulting in aqueous drug dispersions of ∼300 mg·ml^−1^, which surpassed the loading capacity of liposomes prepared using conventional sonication methods (20 mol%) (Kastner *et al.*, 2015). SHM-based devices were also adopted by Joshi *et al.* to manufacture liposomes containing a combination of hydrophilic and lipophilic drugs, metformin, and glipizide, respectively, facilitating their co-delivery (Joshi *et al.*, 2016). Guimarães Sá Correia *et al.* compared the quality and properties of liposomes prepared using the SHM system with liposomes generated through the film hydration method, highlighting the benefits of microfluidics for industrial liposomes production. When compared with conventional liposomes, microfluidics also allowed producing liposomes with smaller particle size by adjusting the above-mentioned parameters TFR and FRR. The NA platform also simplified the drug encapsulation step without compromising efficiency and significantly reduced the manufacturing time compared to traditional methods. Notably, high encapsulation efficacies of hydrophilic atenolol (AT) and lipophilic quinine (Q) compounds, reaching 90% and 88%, respectively, were achieved (Guimarães Sá Correia *et al.*, 2017). Molinaro *et al.* developed a continuous SHM-based process for incorporating membrane proteins derived from leukocytes within the lipid bilayer of liposome-like NPs. These engineered liposomes exhibited intrinsic biological properties, such as delaying macrophage uptake and targeting of inflamed endothelium, as validated through *in vitro* and *in vivo* models (Molinaro *et al.*, 2018). SHM strategies were also employed to formulate different liposomal formulations of curcumin (Lipo-Cur), with variation in factors such as lipid composition, method of fabrication, mixing speed, and solvent ratio [Fig. 1(c)]. Lipo-Cur exhibited increased water solubility of curcumin from 0.4 to ∼280 μg/mL and showed antitumor efficacy when administrated in combination with cisplatin in tumor-bearing mice (Hamano *et al.*, 2019). Recently, Forbes *et al.* introduced in-line purification and at-line monitoring of particle size to enable the efficient and fast manufacture of liposomes incorporating proteins achieving uniform protein loading ranging from 20% to 35%, surpassing the levels from sonication or extrusion methods, typically yielding less than 5%. Importantly, the high loading obtained was a direct result of the production process and was not influenced by the type and concentration of lipids used across the range tested (Forbes *et al.*, 2019).

## BAFFLE MICROMIXER

Although SHM devices show good size controllability, liposomes easily clog chaotic mixers, which can hinder sample flow and productivity.

For this reason, Kimura *et al.* introduced a novel device and named it the invasive lipid nanoparticle production (iLiNP) [Fig. 1(d)]. iLiNP utilizes a two-dimensional baffle mixer design enabling precise size adjustment of liposomes at 10 nm intervals within the 20–100 nm range. Moreover, the iLiNP platform facilitated fabrication of pH-sensitive lipid-siRNA nanocomplexes, which exhibited high gene delivery efficiency, achieving a 90% knockdown of the target gene *in vivo* at a low siRNA dose (Kimura *et al.*, 2018). In a comparative study with the ethanol injection method, the iLiNP device demonstrated superior performance. Compared to the traditional method when encapsulating coenzyme Q10 (CoQ_10_) into liposome for mitochondrial delivery, the iLiNP produced smaller-sized particles and processed larger volumes in shorter times, overcoming scalability challenges. Furthermore, CoQ_10_-loaded liposomes exhibited efficient internalization by HeLa cells, effectively reaching mitochondria. This outcome highlights the potential of the iLiNP device in the field of mitochondrial nanomedicine (Hibino *et al.*, 2019).

## MICROFLUIDIC-BASED ENGINEERED EVS AND EVS-MIMETICS PRODUCTION

EVs and EVs-mimetics have recently garnered significant attention as potential bioinspired DDSs (Herrmann *et al.*, 2021). Generally, modifying EVs and EVs-mimetics typically relies on well-established cell manipulation techniques such as electroporation, sonication, and incubation (Théry *et al.*, 2018). However, these methods often suffer from batch-to-batch variations and limited control over the process, highlighting the need for improved, rapid, automated, and cost-effective engineering technologies in both basic research and clinical applications (Wiklander *et al.*, 2019).

**FIG. 2.**
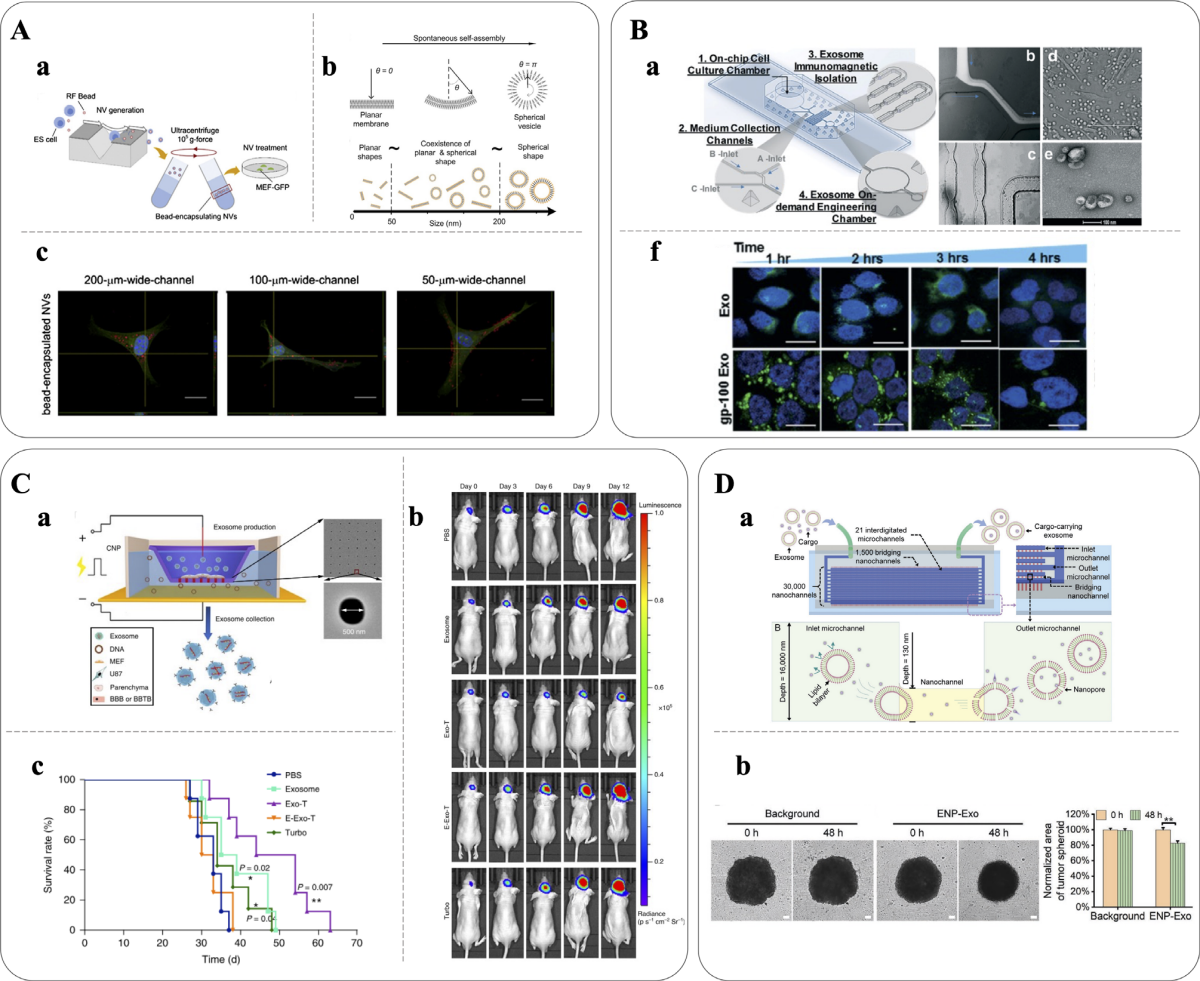
Microfluidic-based production of engineered EVs. (A-a) and (A-b) Schematic of the microfluidic system for the generation of self-assembled nanovesicles by slicing cells with 500 nm-thick silicon nitride blades. During self-assembly, the plasma membrane fragments envelop exogenous materials (here, fluorescent polystyrene beads) that is then successfully delivered across the plasma membrane of recipient cells (MEF-GFP, (A-c); scale bars: 20 *μ*m [Yoon *et al.*, Biomaterials, **59**, 12–20 (2015). Copyright 2015 Authors, licensed under a Creative Commons Attribution (CC BY) license]. (b-a) Schematic of the 3D molded microfluidic device enabling real-time harvesting, antigenic modification (e.g., gp-100), and subsequent photo-release of engineered antigenic EVs. A fluorescent solution was used to demonstrate the flow of immunomagnetic beads mixing with cell culture media (B-b), while bright-field microscopy images showed the serpentine microchannel (B-c) and the on-chip cultured leukocytes (B-d); and (B-e) SEM image of the engineered EVs, visible in (f) labeled with green membrane dye PKH67 in confocal images showing that gp100 enhanced cellular uptake from dendritic monocytes by ∼2 fold compared to native EVs; scale bars: 5 *μ*m. Reproduced with permission from Zhao *et al.*, Lab Chip **19**(10), 1877–1886 (2019). Copyright 2019 Royal Society of Chemistry. (c-a) Nanoporation device for the generation of EVs for targeted nucleic acid delivery. EVs containing PTEN mRNA (Exo-T) were used to treat PTEN-deficient glioma models: mice treated with Exo-Ts showed inhibited tumor growth (C-b) and prolonged survival (C-c) compared with non-targeted EVs (exosomes), empty EVs (E-Exo-T), TurboFect nanoparticles (Turbo), or PBS. Reproduced with permission from Armstrong *et al.*, Nat. Biomed. Eng. **4**(1), 69–83 (2020). Copyright 2019 Springer Nature Limited. (D-a) Schematic of a device to prepare DOX-carrying EVs via generation of membrane nanopores by mechanical compression; (D-b) the effect of DOX-EVs was analyzed using a tumor spheroid model after 48 h incubation: DOX-EVs treated spheroids measured a 17% decrease in cross section, indicating an EVs-mediated inhibitory effect on tumor growth; scale bars: 100 *μ*m. Reproduced with permission from Hao *et al.*, Small **17**, 2102150 (2021). Copyright 2021 Wiley-VCH GmbH.

Microfluidic technologies have emerged as powerful tools for reproducible and high-throughput engineering of EVs, offering improved cargo loading efficiencies, isolation, molecular analysis, and detection (Tian *et al.*, 2022). Given the relatively recent emergence of EVs and EVs-mimetics in therapeutic applications, there has been limited exploration of their engineering using microfluidic technologies, leaving untapped potential to be uncovered. In this section, state-of-the-art microfluidic approaches for producing EVs and EVs-mimetics for DDSs applications are reviewed, including microfluidic extrusion, surface engineering, sonication, electroporation, nanoporation, and mixing; significant examples are reported in Fig. 2.

## MICROFLUIDIC EXTRUSION

Jo *et al.* introduced a novel microfluidic device featuring constriction microchannels for mechanical fractionation of cells into CDNs. They were successful in generating CDNs from embryonic stem cells, with controllable size ranging from 60 to 120 nm and containing essential components such as RNAs and intracellular proteins. The engineered CDNs demonstrated a delivery capacity comparable to naturally secreted EVs, proved by the similar gene expression levels in recipient cells (Jo *et al.*, 2014).

Park's group further optimized the device for CDN production by incorporating an array of 500 nm-thick silicon nitride blades [Fig. 2(a)] that precisely sliced cell membranes as they passed through the device. The resulting sliced cell fragments self-reassembled into CDNs, driven by the minimization of the free energy associated with lipid bilayers. These reassembled CDNs retained both the cellular contents of the source cells and exogenous materials. In a validation experiment with fluorescent microspheres, ∼30% of the particles were successfully encapsulated during reassembly process. Notably, the device enhanced the throughput compared to EVs secreted from an equivalent number of cells, with the number of produced vesicles being ∼100 times higher (Yoon *et al.*, 2015).

## MICROFLUIDIC SURFACE ENGINEERING

In contrast to extrusion techniques that rely on random reassembly of CDNs, surface engineering emerges as a promising strategy to produce EVs secreted from living cells with minimal impairment of their native properties while exploiting the reviewed advantages of microfluidics over conventional benchtop surface engineering methods.

In a study by Zhao *et al.*, a microfluidic platform was developed that integrated on-chip cell culture and streamlined the capture and surface engineering of EVs [Fig. 2(b)]. To selectively capture MHC-1-positive EVs secreted from the on-chip cell culture, the device employed magnetic NPs functionalized with a photo-cleavable linker. Following capture, the immobilized EVs were engineered with surface tumor antigenic peptides. Notably, the engineered EVs exhibited an enhanced internalization capability by antigen-presenting cells compared to native EVs when released through a photo-induced process, thereby facilitating downstream immune stimulation (Zhao *et al.*, 2019).

## MICROFLUIDIC SONICATION

Sonication is an active method for loading cargos into the lumens of EVs by applying acoustic force to deform and permeabilize the membrane. Microfluidic sonication offers distinct advantages, including the one-step formation of membrane-coated NPs with high efficiency in both coating and loading active compounds.

Liu *et al.* introduced a microfluidic sonication platform for the streamlined fabrication of polymeric NPs coated with EV membranes, simultaneously encapsulating imaging agents. This approach effectively reduced the immune clearance of NPs and enhanced their tumor-specific targeting. In this process, a solution containing green fluorescent DiO-labeled poly(lactic-co-glycolic acid) (PLGA) NPs and an EV suspension were co-injected into the microfluidic device, which was then immersed in an ultrasonic bath. The rapid mixing of PLGA with PBS within the microchannel resulted in efficient precipitation of PLGA NPs. Simultaneously, the ultrasonic waves generated intense acoustic pressure, causing the breakage of EV membrane, which swiftly re-assembled around PLGA NPs, forming core-shell structures within a very short time frame (<30 ms). The resulting NPs exhibited reduced nonspecific uptake and enhanced tumor targeting, as demonstrated in both *in vitro* and *in vivo* models, in comparison to lipid-coated NPs of similar sizes (Liu *et al.*, 2019). Recently, the platform was employed to fabricate EV membrane-coated zeolitic imidazolate framework-8 (ZIF-8) NPs loaded with rhodamine B (RhB) for *in situ* imaging of cellular adenosine triphosphate (ATP) (Lv *et al.*, 2021).

## MICROFLUIDIC ELECTROPORATION

Electroporation is another active method employed to load cargo into EVs by creating transient pores on membranes using electric fields. Lee's group developed a microfluidic electroporation device designed for large-scale production of EVs that carry therapeutic mRNAs. The system consisted of a monolayer of source cells such as mouse embryonic fibroblasts and dendritic cells cultured over the platform surface, which contains an array of nanochannels (∼500 nm in diameter) [Fig. 2(c)]. In this device, DNA plasmids were delivered from the buffer to the cells through the nanochannels using transient electrical pulses. The transfected cells were then able to release EVs containing functional mRNAs when a voltage was applied across the nanochannels. Compared to conventional electroporation methods, this system demonstrated a 50-fold increase in EVs production and an over 10^3^-fold increase in the loading efficiency of mRNAs, resulting in 2 − 10 intact mRNAs per EV. Finally, the engineered EVs containing PTEN mRNA exhibited restoration of tumor-suppressor function and prolonged the survival of mice with PTEN-deficient brain gliomas (Yang *et al.*, 2020).

## MICROFLUIDIC NANOPORATION

Nanoporation is a technique that enables the introduction of cargo into EVs by transporting it through nanochannels to permeabilize their membranes. Recently, Hao *et al.* introduced a nanofluidic platform named exosome nanoporator (ENP) that allows direct cargo loading into EVs through mechanical compression and fluid shear [Fig. 2(d)]. The ENP consists of a PDMS layer containing interconnected microchannels bonded to a glass layer with an array of 3 × 10^4^ perpendicular nanochannels. When the height of nanochannels is close to the diameter of EVs, their membranes experience mechanical compression inducing permeabilization and allowing encapsulation of cargo such as DOX while maintaining EVs' integrity. The EVs generated using the ENP successfully delivered their cargo to human non-small cell lung cancer cells, leading to cell death (Hao *et al.*, 2021).

## MICROFLUIDIC MIXING

In a recent study, Tokeshi's group used their iLiNP microfluidic device to generate synthetic EV-mimetics NPs that efficiently encapsulated siRNAs while also providing control over the NP size by adjusting flow conditions. Interestingly, the siRNA-loaded artificial EVs exhibited the ability to transfect cells, resulting in effective gene-silencing activity and evidencing the potential of this approach in designing functional artificial EVs (Kimura *et al.*, 2021).

## CONCLUSIONS

The effective delivery of therapeutic agents to their intended targets is a crucial aspect of drug design and development. Indeed, the ultimate objective of every DDS is to efficiently achieve clinical application and tangible benefits for patients (Kooijmans *et al.*, 2012). Lipid-based nanoparticles, such as liposomes and EVs, have emerged as promising carriers for delivering biological therapeutics to the cytosol of target cells.

Although liposomes are commonly used for clinical delivery of various therapeutic agents including small molecules, proteins, and RNAs, the full potential of nano DDSs requires the development of more complex and multifunctional devices.

Designing optimal DDSs necessitates the incorporation of complexity like that of the biological environment to overcome challenges such as clearance, degradation, and physical barriers. Moreover, strategies for controlled and site-specific drug delivery are crucial for ensuring desired therapeutic effects.

The use of EVs as DDSs holds promise in overcoming several challenges in nanomedicine, first thanks to their high organotropism. However, their complexity and difficult isolation and purification represent obstacles for proper clinical translation. One alternative approach could be the synthetic assembly of liposomes that mimic the crucial components of natural EVs. By focusing on the essential components responsible for the organotropism, functional synthetic EVs-mimetics can be created. However, collaboration between the fields of synthetic nanomedicine and EVs research would be the key to provide valuable insights and solutions for the successful development of artificial EVs-mimetics (Wolfram and Ferrari, 2019).

In addition to using synthetic materials, EVs-mimetics have been developed using other physical-origin sources as starting materials, with a prominent example being whole cells. These CDNs inherit the surface properties of their parent cells, making them highly biocompatible and endowed with inherent targeting capabilities. Moreover, compared to traditional methods for isolating EVs, the fabrication techniques for CDNs hold significant advantages, allowing for the production of larger quantities of nanovesicles in a shorter timeframe (Jang *et al.*, 2013).

Generally, the primary challenge for NPs fabrication remains the need to enhance production yield and improving drug encapsulation efficiency. Microfluidic technology could facilitate research in NP fabrication for drug delivery applications by offering versatility, reproducibility, and scalability, in the high-throughput production of liposomes, and high-resolution manipulation of EVs and EVs-mimetics for clinical applications. Additionally, microfluidic devices serve as valuable tools for drug screening through cell-on-a-chip, organ-on-a-chip, and human-on-a-chip platforms, which can assess drug responses and partially reduce the reliance on animal models in research (Ronaldson-Bouchard *et al.*, 2022).

The integration of microfluidic systems with EVs isolation, engineering, and cell culture represent a promising research direction for advancing therapeutic delivery mediated by EVs. This interdisciplinary approach is expected to contribute to the development of novel therapeutic strategies and usher in a new era in precision medicine in the coming years.

## Data Availability

Data sharing is not applicable to this article as no new data were created or analyzed in this study.
